# Predictive value of radiomics for intracranial aneurysm rupture: a systematic review and meta-analysis

**DOI:** 10.3389/fnins.2024.1474780

**Published:** 2024-10-09

**Authors:** Haoda Wang, Haidong Xu, Junsheng Fan, Jie Liu, Liangfu Li, Zailiang Kong, Hui Zhao

**Affiliations:** ^1^Department of Radiology, The First Hospital of Huhhot, Huhhot, China; ^2^Department of Radiotherapy, Affiliated Hospital of Inner Mongolia Medical University, Huhhot, China

**Keywords:** radiomics, intracranial aneurysm rupture, meta-analysis, systematic review, diagnosis

## Abstract

**Objective:**

To systematically review the literature on radiomics for predicting intracranial aneurysm rupture and conduct a meta-analysis to obtain evidence confirming the value of radiomics in this prediction.

**Methods:**

A systematic literature search was conducted in PubMed, Web of Science, Embase, and The Cochrane Library databases up to March 2024. The QUADAS-2 tool was used to assess study quality. Stata 15.0 and Review Manager 5.4.1 were used for statistical analysis. Outcomes included combined sensitivity (Sen), specificity (Spe), positive likelihood ratio (+LR), negative likelihood ratio (−LR), diagnostic odds ratio (DOR), and their 95% confidence intervals (CI), as well as pre-test and post-test probabilities. The SROC curve was plotted, and the area under the curve (AUC) was calculated. Publication bias and small-study effects were assessed using the Deeks’ funnel plot.

**Results:**

The 9 included studies reported 4,284 patients, with 1,411 patients with intracranial aneurysm rupture (prevalence 32.9%). The overall performance of radiomics for predicting intracranial aneurysm rupture showed a combined Sen of 0.78 (95% CI: 0.74–0.82), Spe of 0.74 (95% CI: 0.70–0.78), +LR of 3.0 (95% CI: 2.7–3.4), −LR of 0.29 (95% CI: 0.25–0.35), DOR of 10 (95% CI: 9–12), and AUC of 0.83 (95% CI: 0.79–0.86). Significant heterogeneity was observed in both Sen (I^2^ = 90.93, 95% CI: 89.00–92.87%) and Spe (I^2^ = 94.28, 95% CI: 93.21–95.34%).

**Conclusion:**

Radiomics can improve the diagnostic efficacy of intracranial aneurysm rupture. More large-sample, prospective, multicenter clinical studies are needed to further evaluate its predictive value.

**Systematic review registration:**

https://www.crd.york.ac.uk/PROSPERO/.

## 1 Introduction

Intracranial aneurysm is a tumor-like protrusion formed when the lumen of an intracranial artery gradually expands and bulges due to localized damage to the arterial wall caused by congenital developmental abnormalities or acquired injuries, under the influence of hemodynamic load or other factors (Etminan and Rinkel, 2016). With the development of imaging technology, the detection rate of unruptured intracranial aneurysms is increasing annually (Greving et al., 2014). However, the treatment decision for unruptured intracranial aneurysms remains controversial. Many intracranial aneurysms are asymptomatic and do not rupture for an extended period (Korja et al., 2014). Currently, preventive treatment for unruptured intracranial aneurysms includes endovascular treatment and neurosurgical interventional treatment, both associated with the inevitable risk of treatment-related complications (Naggara et al., 2010; Zhu et al., 2020). Conversely, once an intracranial aneurysm ruptures, it leads to subarachnoid hemorrhage (SAH), a severe subtype of stroke often accompanied by high mortality and disability rates (Vlak et al., 2011). Therefore, early screening of patients with intracranial aneurysms at high risk of rupture and providing them with timely preventive treatment are crucial for improving patient prognosis.

Previous studies have demonstrated that the risk of intracranial aneurysm rupture is associated with its morphological characteristics (Lindgren et al., 2016). Researchers have measured and defined these characteristics, such as neck width, aneurysm angle, and height, on computed tomographic angiography (CTA) or digital subtraction angiography (DSA) images of intracranial aneurysms (Zhang et al., 2019; Chen et al., 2020). Radiomics, a new computer-assisted technology, extracts quantitative features (e.g., shape, intensity, and texture) from biomedical images in an objective, reproducible, and high-throughput manner (Zhou et al., 2018; Hua et al., 2020; Tomaszewski and Gillies, 2021). The principle is that pathophysiological changes of certain diseases are manifested in digital medical images, and radiomics can extract this information through quantitative analysis and subsequent biological information mining (Gillies et al., 2016). Recently, the application of radiomics in cerebrovascular diseases has increased (Chen et al., 2021; Zhu et al., 2021; Zhu et al., 2021). Some studies have demonstrated an association between radiomics characteristics of aneurysms and the risk of intracranial aneurysm rupture (Liu et al., 2019; Ou et al., 2021). However, these studies did not analyze the stability of the imaging features, which can be affected by differences in scanning parameters or image segmentation methods (Mackin et al., 2015; Choe et al., 2019). Furthermore, most previous studies on imaging features of intracranial aneurysms have not been validated by external datasets, leaving the reproducibility and generalizability of their results uncertain (Zhu et al., 2021; Luo et al., 2023; Turhon et al., 2023).

Recently, the rapid development of computer science, particularly the progress of artificial intelligence, has led to algorithms such as machine learning and deep learning playing an increasingly important role in medical data processing (Chen et al., 2021). Algorithms, including support vector machines, random forests, and artificial neural networks, have been successfully applied to disease diagnosis, metastasis prediction, and treatment prognosis evaluation (Orru et al., 2012; Beig et al., 2019; Khorrami et al., 2019). Appropriate algorithms can maximize the mining of data information and assist in disease diagnosis. To date, many radiomic models have been used to predict ruptured intracranial aneurysms. However, no relevant meta-analysis has been reported, and its sensitivity and specificity remain undetermined. Therefore, this study systematically searched Pubmed, Embase, Cochrane and Web of Science to obtain all literatures on radiomics in predicting intracranial aneurysm rupture, and conducted a meta-analysis for the first time to obtain the latest and most comprehensive evidence-based confirmation of the value of radiomics in predicting intracranial aneurysm rupture.

## 2 Methods

### 2.1 Protocol and registration

This systematic review and meta-analysis, prospectively registered in PROSPERO (CRD42024453092), was performed following the PRISMA 2020 statement (Page et al., 2021).

### 2.2 Search strategy

A systematic literature search was conducted in PubMed, Web of Science, Embase, and The Cochrane Library databases using medical subject headings and free words, including “radiomics,” “aneurysm,” “rupture,” and “intracranial.” The search strategies for PubMed were as follows: ((“Intracranial Aneurysm”[Mesh]) OR (((((((((((((((((((((((((((((((((((((Aneurysms, Intracranial) OR (Intracranial Aneurysms)) OR (Aneurysm, Intracranial)) OR (Aneurysm, Anterior Communicating Artery)) OR (Anterior Communicating Artery Aneurysm)) OR (Aneurysm, Basilar Artery)) OR (Aneurysms, Basilar Artery)) OR (Artery Aneurysm, Basilar)) OR (Artery Aneurysms, Basilar)) OR (Basilar Artery Aneurysms)) OR (Basilar Artery Aneurysm)) OR (Aneurysm, Middle Cerebral Artery)) OR (Middle Cerebral Artery Aneurysm)) OR (Aneurysm, Posterior Cerebral Artery)) OR (Posterior Cerebral Artery Aneurysm)) OR (Berry Aneurysm)) OR (Aneurysm, Berry)) OR (Aneurysms, Berry)) OR (Berry Aneurysms)) OR (Brain Aneurysm)) OR (Aneurysm, Brain)) OR (Aneurysms, Brain)) OR (Brain Aneurysms)) OR (Cerebral Aneurysm)) OR (Aneurysms, Cerebral)) OR (Cerebral Aneurysms)) OR (Aneurysm, Cerebral)) OR (Giant Intracranial Aneurysm)) OR (Aneurysm, Giant Intracranial)) OR (Aneurysms, Giant Intracranial)) OR (Giant Intracranial Aneurysms)) OR (Intracranial Aneurysm, Giant)) OR (Intracranial Aneurysms, Giant)) OR (Aneurysm, Anterior Cerebral Artery)) OR (Anterior Cerebral Artery Aneurysm)) OR (Aneurysm, Posterior Communicating Artery)) OR (Posterior Communicating Artery Aneurysm))) AND (((radiomic*) OR (Histogram*)) OR (Texture*)). The search time was up to March, 2024. Furthermore, we manually screened the bibliography lists of all included studies. Two authors retrieved and assessed eligible articles independently. Any differences in literature retrieval were resolved by discussion. All retrieved literature was manually reviewed and verified through EndNote X9.

### 2.3 Inclusion and exclusion criteria

This study included prospective and retrospective studies investigating the predictive value of radiomics for intracranial aneurysm rupture. Inclusion criteria:

P: patients with suspected or confirmed ruptured intracranial aneurysms.

I: radiomics as the evaluated experiment, regardless of model and method.

C: gold standard, including CTA, MRA, or DSA.

O: sensitivity (Sen), specificity (Spe), positive likelihood ratio (+LR), negative likelihood ratio (−LR), diagnostic odds ratio (DOR), pre-test probability, post-test probability, summary receiver operating characteristic (SROC) area under the curve (AUC).

S: prospective and retrospective studies focusing on the predictive value of radiomics for intracranial aneurysm rupture.

Exclusion criteria: (Etminan and Rinkel, 2016) duplicate publications; (Greving et al., 2014) irrelevant articles; (Korja et al., 2014) reviews, meta-analyses, conferences, letters, comments; (Naggara et al., 2010) animal models, studies with low sample size; (Zhu et al., 2020) articles with no or incomplete data; (Vlak et al., 2011) non-English articles.

### 2.4 Data extraction

Two independent reviewers extracted data from eligible studies, including first author, publication year, study design, subjects’ age, gender, radiomics models, gold standard, sample size of case and control groups, true positives (TP), false positives (FP), true negatives (TN), false negatives (FN), Sen, Spe, +LR, −LR, DOR, pre-test probability, post-test probability, and AUC value. Disagreements between the two reviewers were resolved by consulting a third party. Corresponding authors were contacted for full data if research data was insufficient. All data were summarized in a Microsoft Excel spreadsheet.

### 2.5 Quality assessment

The quality assessment of the included literature was conducted using the QUADAS-2 tool, following the guidelines in the Cochrane Handbook for systematic reviews of diagnostic trials. The final evaluation findings were presented using the Review Manager 5.4.1 software (Qu et al., 2018). Two reviewers independently assessed the literature quality, with disagreements resolved through discussion or adjudication by a third reviewer when necessary. The QUADAS-2 tool assesses literature quality from two aspects: risk of bias and clinical applicability. The risk of bias comprises four aspects: patient selection, index test, reference standard, and flow and timing. The clinical applicability questions consist of three aspects: patient selection, index test, and reference standard. Each evaluation domain in the risk of bias contains signaling questions with “yes/no/unclear” answer options. If all signaling questions in a domain are answered “yes,” the risk of bias in that aspect is considered low. If one answer is “no,” there is a possibility of bias. Clinical applicability has no signaling questions, only an overall assessment with “high risk/low risk/unclear” answer options. The “unclear” option can only be selected when the literature provides incomplete information during the assessment process. In addition, the Radiomics Quality Score (RQS) was used to assess the characteristics and quality of each included radiomics study. This scoring scale has 36 potential scores based on 16 criteria, with 36 indicating the highest quality (Spadarella et al., 2023).

### 2.6 Data analysis

Stata 15.0 and Review Manager 5.4.1 software were used for statistical analysis. A bivariate mixed-effects regression model was employed after the random-effects model to combine Sen, Spe, +LR, −LR, DOR, and their 95% confidence intervals (95% CI), as well as pre-test and post-test probabilities. The SROC curve was plotted, and the AUC was calculated. The *χ*^2^ test, with a test level of *α* = 0.05, was used to analyze the statistical heterogeneity among studies, and the *I*^2^ value quantitatively determined the heterogeneity. An I^2^ ≤ 50% indicated low heterogeneity, while a higher value suggested high heterogeneity. The Deeks’ funnel plot assessed publication bias and assumed small-study effects, with *p* < 0.05 indicating publication bias in the included literature. When publication bias is present, the trim-and-fill method is used to assess the effect of the bias on the combined statistics (Zhang and Zhong, 2008).

## 3 Results

### 3.1 Literature screening process and results

**Figure 1:**
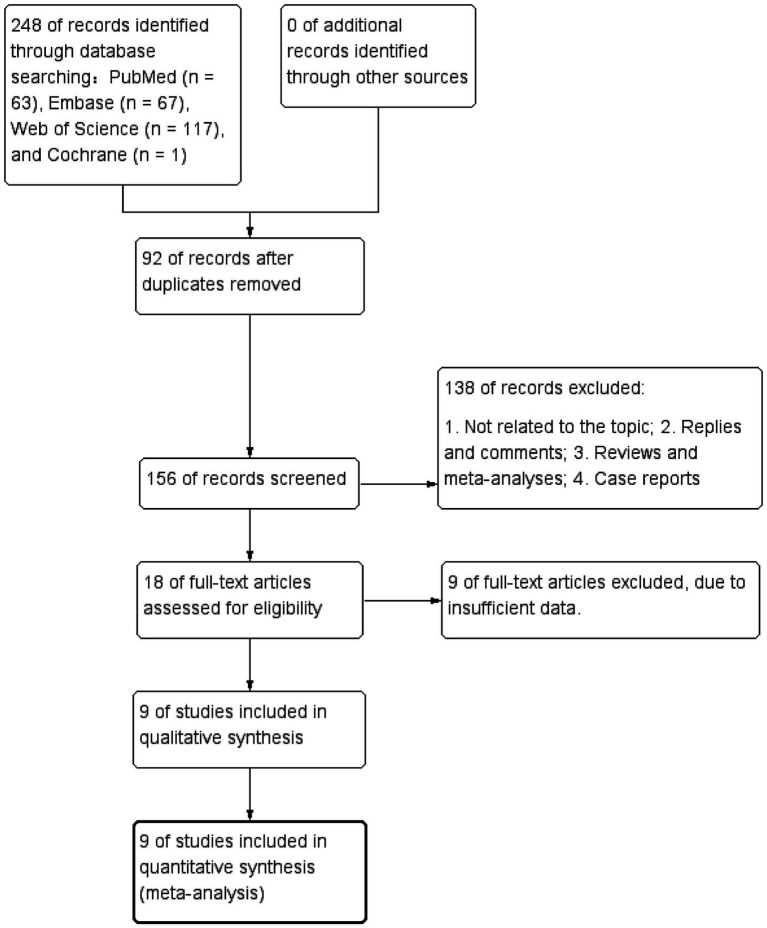
PRISMA flow-chart for the systematic review and meta-analysis.

A total of 248 articles were obtained using the literature search strategy. After removing duplicates using EndNote X9, 156 articles remained. Following the exclusion of 138 articles based on titles and abstracts, 18 articles underwent initial screening. Among these, 9 articles were further excluded after full-text review due to incomplete information and not meeting the requirements, resulting in a final inclusion of 9 articles (Ou et al., 2021; Alwalid et al., 2021; Zhu et al., 2021; Li et al., 2022; Ou et al., 2022; Yamanouchi et al., 2022; Luo et al., 2023; Turhon et al., 2023; Yang et al., 2023). Figure 1 illustrates the literature screening process flowchart, and Table 1 presents the fundamental characteristics of the included studies. For studies with multiple models or cohorts, separate extraction was performed, distinguished by adding the letters “a, b, c, or d” after the study names.

**Table 1:** Characteristic of included studies.

Author	Year	Cohort	Region	Study design	Population	Feature extraction soft
Alwalid	2021a	Training	China	Prospective/retrospective	(1) Adult patient over 18 years old and (2) a diagnosis of intracranial aneurysm on CT angiography regardless of the rupture status of the aneurysm.	Pyradiomics package
Alwalid	2021b	Test	China	Prospective/retrospective	(1) Adult patient over 18 years old and (2) a diagnosis of intracranial aneurysm on CT angiography regardless of the rupture status of the aneurysm.	Pyradiomics package
Li	2022a	training	China	Prospective	(1) confirmed diagnosis of one or more unruptured saccular cerebral aneurysms at baseline by CTA (positive diagnosis in the clinical record by experienced radiologist); (2) aneurysm maximum diameter > 2 mm and lesion morphology that is differentiable from infundibulum; (3) image quality allowed detailed depiction of aneurysm morphology and parent vessels, and was sufficient for the segmentation without substantial artifacts.	Pyradiomics package
Li	2022b	test	China	Prospective	(1) confirmed diagnosis of one or more unruptured saccular cerebral aneurysms at baseline by CTA (positive diagnosis in the clinical record by experienced radiologist); (2) aneurysm maximum diameter > 2 mm and lesion morphology that is differentiable from infundibulum; (3) image quality allowed detailed depiction of aneurysm morphology and parent vessels, and was sufficient for the segmentation without substantial artifacts.	Pyradiomics package
Li	2022c	training	China	Prospective	(1) confirmed diagnosis of one or more unruptured saccular cerebral aneurysms at baseline by CTA (positive diagnosis in the clinical record by experienced radiologist); (2) aneurysm maximum diameter > 2 mm and lesion morphology that is differentiable from infundibulum; (3) image quality allowed detailed depiction of aneurysm morphology and parent vessels, and was sufficient for the segmentation without substantial artifacts.	Pyradiomics package
Li	2022d	test	China	Prospective	(1) confirmed diagnosis of one or more unruptured saccular cerebral aneurysms at baseline by CTA (positive diagnosis in the clinical record by experienced radiologist); (2) aneurysm maximum diameter > 2 mm and lesion morphology that is differentiable from infundibulum; (3) image quality allowed detailed depiction of aneurysm morphology and parent vessels, and was sufficient for the segmentation without substantial artifacts.	Pyradiomics package
Luo	2023a	Internal	China	Retrospective	(1) ruptured or unruptured saccular CAs located in the anterior circulation arteries and, the subarachnoid hemorrhage can be explicitly attributed to aneurysm rupture; (2) a minimum aneurysm diameter > 3 mm; and (3) good- quality cerebrovascular imaging—CTA or MRA data—that can adequately be used to reconstruct the aneurysm and 3–5 mm of its adjacent parent arteries with clear boundaries.	Pyradiomics package
Luo	2023b	Internal	China	Retrospective	(1) ruptured or unruptured saccular CAs located in the anterior circulation arteries and, the subarachnoid hemorrhage can be explicitly attributed to aneurysm rupture; (2) a minimum aneurysm diameter > 3 mm; and (3) good- quality cerebrovascular imaging—CTA or MRA data—that can adequately be used to reconstruct the aneurysm and 3–5 mm of its adjacent parent arteries with clear boundaries.	Pyradiomics package
Luo	2023c	Internal	China	Retrospective	(1) ruptured or unruptured saccular CAs located in the anterior circulation arteries and, the subarachnoid hemorrhage can be explicitly attributed to aneurysm rupture; (2) a minimum aneurysm diameter > 3 mm; and (3) good- quality cerebrovascular imaging—CTA or MRA data—that can adequately be used to reconstruct the aneurysm and 3–6 mm of its adjacent parent arteries with clear boundaries.	Pyradiomics package
Luo	2023d	External	China	Retrospective	(1) ruptured or unruptured saccular CAs located in the anterior circulation arteries and, the subarachnoid hemorrhage can be explicitly attributed to aneurysm rupture; (2) a minimum aneurysm diameter > 3 mm; and (3) good- quality cerebrovascular imaging—CTA or MRA data—that can adequately be used to reconstruct the aneurysm and 3–7 mm of its adjacent parent arteries with clear boundaries.	Pyradiomics package
Luo	2023e	External	China	Retrospective	(1) ruptured or unruptured saccular CAs located in the anterior circulation arteries and, the subarachnoid hemorrhage can be explicitly attributed to aneurysm rupture; (2) a minimum aneurysm diameter > 3 mm; and (3) good- quality cerebrovascular imaging—CTA or MRA data—that can adequately be used to reconstruct the aneurysm and 3–8 mm of its adjacent parent arteries with clear boundaries.	Pyradiomics package
Luo	2023f	External	China	Retrospective	(1) ruptured or unruptured saccular CAs located in the anterior circulation arteries and, the subarachnoid hemorrhage can be explicitly attributed to aneurysm rupture; (2) a minimum aneurysm diameter > 3 mm; and (3) good- quality cerebrovascular imaging—CTA or MRA data—that can adequately be used to reconstruct the aneurysm and 3–9 mm of its adjacent parent arteries with clear boundaries.	Pyradiomics package
Ou	2021a		China	Prospective	A confirmed diagnosis of one or more intracranial aneurysms	Pyradiomics package
Ou	2021b		China	Prospective	A confirmed diagnosis of one or more intracranial aneurysms	Pyradiomics package
Ou	2022		China	Retrospective	A confirmed diagnosis of one or more intracranial aneurysms	Pyradiomics package
Turhon	2023a	Trainin	China	Retrospective	(1) at least one intracranial aneurysm confirmed by digital subtraction angiography (DSA) imaging; (2) older than 18 years; and (3) accessibility of morphological and radiomics data.	Pyradiomics package
Turhon	2023b	Trainin	China	Retrospective	(1) at least one intracranial aneurysm confirmed by digital subtraction angiography (DSA) imaging; (2) older than 18 years; and (4) accessibility of morphological and radiomics data.	Pyradiomics package
Turhon	2023c	Trainin	China	Retrospective	(1) at least one intracranial aneurysm confirmed by digital subtraction angiography (DSA) imaging; (2) older than 18 years; and (5) accessibility of morphological and radiomics data.	Pyradiomics package
Turhon	2023d	Internal validatio	China	Retrospective	(1) at least one intracranial aneurysm confirmed by digital subtraction angiography (DSA) imaging; (2) older than 18 years; and (6) accessibility of morphological and radiomics data.	Pyradiomics package
Turhon	2023e	Internal validatio	China	Retrospective	(1) at least one intracranial aneurysm confirmed by digital subtraction angiography (DSA) imaging; (2) older than 18 years; and (7) accessibility of morphological and radiomics data.	Pyradiomics package
Turhon	2023f	Internal validatio	China	Retrospective	(1) at least one intracranial aneurysm confirmed by digital subtraction angiography (DSA) imaging; (2) older than 18 years; and (8) accessibility of morphological and radiomics data.	Pyradiomics package
Turhon	2023 g	External validation	China	Retrospective	(1) at least one intracranial aneurysm confirmed by digital subtraction angiography (DSA) imaging; (2) older than 18 years; and (9) accessibility of morphological and radiomics data.	Pyradiomics package
Turhon	2023 h	External validation	China	Retrospective	(1) at least one intracranial aneurysm confirmed by digital subtraction angiography (DSA) imaging; (2) older than 18 years; and (10) accessibility of morphological and radiomics data.	Pyradiomics package
Turhon	2023i	External validation	China	Retrospective	(1) at least one intracranial aneurysm confirmed by digital subtraction angiography (DSA) imaging; (2) older than 18 years; and (11) accessibility of morphological and radiomics data.	Pyradiomics package
Yamanouchi	2022	Test	Japan	Prospective	Patients who underwent MRA for intracranial aneurysms examination	Pyradiomics package
Yang	2023a	Validation	China	Retrospective	Patients diagnosed with intracranial aneurysms by CTA or digital subtraction angiography (DSA)	Pyradiomics package
Yang	2023b	Validation	China	Retrospective	Patients diagnosed with intracranial aneurysms by CTA or digital subtraction angiography (DSA)	Pyradiomics package
Yang	2023c	Validation	China	Retrospective	Patients diagnosed with intracranial aneurysms by CTA or digital subtraction angiography (DSA)	Pyradiomics package
Yang	2023d	Validation	China	Retrospective	Patients diagnosed with intracranial aneurysms by CTA or digital subtraction angiography (DSA)	Pyradiomics package
Yang	2023e	Validation	China	Retrospective	Patients diagnosed with intracranial aneurysms by CTA or digital subtraction angiography (DSA)	Pyradiomics package
Yang	2023f	Validation	China	Retrospective	Patients diagnosed with intracranial aneurysms by CTA or digital subtraction angiography (DSA)	Pyradiomics package
Yang	2023 g	Validation	China	Retrospective	Patients diagnosed with intracranial aneurysms by CTA or digital subtraction angiography (DSA)	Pyradiomics package
Yang	2023 h	Validation	China	Retrospective	Patients diagnosed with intracranial aneurysms by CTA or digital subtraction angiography (DSA)	Pyradiomics package
Yang	2023i	Validation	China	Retrospective	Patients diagnosed with intracranial aneurysms by CTA or digital subtraction angiography (DSA)	Pyradiomics package
Yang	2023j	Validation	China	Retrospective	Patients diagnosed with intracranial aneurysms by CTA or digital subtraction angiography (DSA)	Pyradiomics package
Yang	2023 k	Validation	China	Retrospective	Patients diagnosed with intracranial aneurysms by CTA or digital subtraction angiography (DSA)	Pyradiomics package
Yang	2023 L	Validation	China	Retrospective	Patients diagnosed with intracranial aneurysms by CTA or digital subtraction angiography (DSA)	Pyradiomics package
Zhu	2021a	Training	China	Retrospective	MCA aneurysms with available computed tomography angiography (CTA) data were included	Pyradiomics package
Zhu	2021b	Training	China	Retrospective	MCA aneurysms with available computed tomography angiography (CTA) data were included	Pyradiomics package
Zhu	2021c	Training	China	Retrospective	MCA aneurysms with available computed tomography angiography (CTA) data were included	Pyradiomics package
Zhu	2021d	Temporal validation dataset	China	Retrospective	MCA aneurysms with available computed tomography angiography (CTA) data were included	Pyradiomics package
Zhu	2021e	Temporal validation dataset	China	Retrospective	MCA aneurysms with available computed tomography angiography (CTA) data were included	Pyradiomics package
Zhu	2021f	Temporal validation dataset	China	Retrospective	MCA aneurysms with available computed tomography angiography (CTA) data were included	Pyradiomics package
Zhu	2021 g	External validation dataset	China	Retrospective	MCA aneurysms with available computed tomography angiography (CTA) data were included	Pyradiomics package
Zhu	2021 h	External validation dataset	China	Retrospective	MCA aneurysms with available computed tomography angiography (CTA) data were included	Pyradiomics package
Zhu	2021i	External validation dataset	China	Retrospective	MCA aneurysms with available computed tomography angiography (CTA) data were included	Pyradiomics package

Author	Model (Machinelearning)	Imaging omics	Gold standard	Clinic patient information					
				Patients		Age		Gender	
				Positive	Negative	Ruptured	Unruptured	Male	Female
Alwalid	LR	1.Wavelet-HHL.firstorder.Entropy, 2.LBP-3D-m1.firstorder.90Percentile, 3.LBP-3D-m2.firstorder.Skewness, 4.LoG-sigma-20 mm-3D.GLCM.ID, 5.LoG-sigma-20 mm-3D.GLSZM.SmallAreaHighGrayLevelEmphasis, 6.LoG-sigma-30 mm-3D.GLCM.InverseVariance, 7.Wavelet-LHH.firstorder.RootMeanSquared, 8.Wavelet-LHL.firstorder.Median, 9.Wavelet-LLH.GLDM.SmallDependenceEmphasis	1.spontaneous subarachnoid hemorrhage, 2.CTA, 3.DSA	152	241	55 ± 9	57 ± 11	159	234
Alwalid	LR	1.Wavelet-HHL.firstorder.Entropy, 2.LBP-3D-m1.firstorder.90Percentile, 3.LBP-3D-m2.firstorder.Skewness, 4.LoG-sigma-20 mm-3D.GLCM.ID, 5.LoG-sigma-20 mm-3D.GLSZM.SmallAreaHighGrayLevelEmphasis, 6.LoG-sigma-30 mm-3D.GLCM.InverseVariance, 7.Wavelet-LHH.firstorder.RootMeanSquared, 8.Wavelet-LHL.firstorder.Median, 10.Wavelet-LLH.GLDM.SmallDependenceEmphasis	1.spontaneous subarachnoid hemorrhage, 2.CTA, 3.DSA	152	241	55 ± 9	57 ± 11	159	234
Li	Radiomics derived features	wavelet.HLL_glcm_Correlation wavelet.HLH_glszm_SizeZoneNonUniformityNormalized original_glszm_SmallAreaLowGrayLevelEmphasis wavelet.HLL_glszm_GrayLevelNonUniformity wavelet.LHL_firstorder_Median wavelet.HHL_gldm_LargeDependenceHighGrayLevelEmphasis	CTA	11	216	61 (49–68)	78	149	
Li	Radiomics derived features	wavelet.HLL_glcm_Correlation wavelet.HLH_glszm_SizeZoneNonUniformityNormalized original_glszm_SmallAreaLowGrayLevelEmphasis wavelet.HLL_glszm_GrayLevelNonUniformity wavelet.LHL_firstorder_Median wavelet.HHL_gldm_LargeDependenceHighGrayLevelEmphasis	CTA	11	216	61 (49–68)	78	149	
Li	Radiomics derived morphological features	wavelet.HLL_glcm_Correlation wavelet.HLH_glszm_SizeZoneNonUniformityNormalized original_glszm_SmallAreaLowGrayLevelEmphasis wavelet.HLL_glszm_GrayLevelNonUniformity wavelet.LHL_firstorder_Median wavelet.HHL_gldm_LargeDependenceHighGrayLevelEmphasis	CTA	11	216	61 (49–68)	78	149	
Li	Radiomics derived morphological features	wavelet.HLL_glcm_Correlation wavelet.HLH_glszm_SizeZoneNonUniformityNormalized original_glszm_SmallAreaLowGrayLevelEmphasis wavelet.HLL_glszm_GrayLevelNonUniformity wavelet.LHL_firstorder_Median wavelet.HHL_gldm_LargeDependenceHighGrayLevelEmphasis	CTA	11	216	61 (49–68)	78	149	
Luo	Ridge regression	/	CTA and MRA	148	270	58.62 ± 12.48	60.99 ± 12.9	164	254
Luo	SVM	/	CTA and MRA	148	270	58.62 ± 12.48	60.99 ± 12.9	164	254
Luo	NN	/	CTA and MRA	148	270	58.62 ± 12.48	60.99 ± 12.9	164	254
Luo	Ridge regression	/	CTA and MRA	105	221	/	/	/	/
Luo	SVM	/	CTA and MRA	105	221	/	/	/	/
Luo	NN	/	CTA and MRA	105	221	/	/	/	/
Ou	Model constructed with addition of shape features derived from radiomics		CTA	29	93	54.20 ± 13.10	55.75 ± 10.37	50	72
Ou	Model constructed with further inclusion offirst-order histogram features and second-order texture features		CTA	29	93	54.20 ± 13.10	55.75 ± 10.37	50	72
Ou	Radiomics with clinical info + LASSO regression		DSA, CTA, and MRA	29	91	54.20 ± 13.10	55.75 ± 10.37	48	72
Turhon	Radiomics morphological feature		DSA	272	885	54.35 ± 12.20	55.50 ± 9.40	394	763
Turhon	Radiomics morphological feature		DSA	272	885	54.35 ± 12.20	55.50 ± 9.40	394	763
Turhon	Radiomics morphological feature		DSA	272	885	54.35 ± 12.20	55.50 ± 9.40	394	763
Turhon	Clinical and radiomics	/	DSA	80	214	55.40 ± 9.73		84	210
Turhon	Clinical and radiomics	/	DSA	80	214	55.40 ± 9.73		84	210
Turhon	Clinical and radiomics	/	DSA	80	214	55.40 ± 9.73		84	210
Turhon	Clinical, morphological, and radiomics		DSA	85	273	56.84 ± 10.52	119	239	
Turhon	Clinical, morphological, and radiomics		DSA	85	273	56.84 ± 10.52	119	239	
Turhon	Clinical, morphological, and radiomics		DSA	85	273	56.84 ± 10.52	119	239	
Yamanouchi	LASSO (Energy.1 (texture/GLCM) + LLH Variance.1 (texture/GLCM) + LLH ZP (texture/GLSZM))		MRA	10	18	35–92 (median: 63)	9	19	
Yang	Random forest	/	CTA and DSA	67	106	58.37 ± 11.76	64.23 ± 13.18	75	98
Yang	Support machine learning		CTA and DSA	67	106	58.37 ± 11.76	64.23 ± 13.18	75	98
Yang	Decision-making tree	/	CTA and DSA	67	106	58.37 ± 11.76	64.23 ± 13.18	75	98
Yang	eXtreme gradient Boosting		CTA and DSA	67	106	58.37 ± 11.76	64.23 ± 13.18	75	98
Yang	Gaussian Naive Bayes	/	CTA and DSA	67	106	58.37 ± 11.76	64.23 ± 13.18	75	98
Yang	Logistic regression	/	CTA and DSA	67	106	58.37 ± 11.76	64.23 ± 13.18	75	98
Yang	K-nearest neighbor	/	CTA and DSA	67	106	58.37 ± 11.76	64.23 ± 13.18	75	98
Yang	Bagging classifier	/	CTA and DSA	67	106	58.37 ± 11.76	64.23 ± 13.18	75	98
Yang	AdaBoost	/	CTA and DSA	67	106	58.37 ± 11.76	64.23 ± 13.18	75	98
Yang	Gradient boosting	/	CTA and DSA	67	106	58.37 ± 11.76	64.23 ± 13.18	75	98
Yang	Light gradient boosting Machine		CTA and DSA	67	106	58.37 ± 11.76	64.23 ± 13.18	75	98
Yang	CatBoost	/	CTA and DSA	67	106	58.37 ± 11.76	64.23 ± 13.18	75	98
Zhu	Radiomics	/	CTA	297	141	55.0 (48.0, 64.3)	59.0 (53.0, 69.0)	188	250
Zhu	Radiomics-morphological		CTA	297	141	55.0 (48.0, 64.3)	59.0 (53.0, 69.0)	188	250
Zhu	Clinical-radiomics-morphological		CTA	297	141	55.0 (48.0, 64.3)	59.0 (53.0, 69.0)	188	250
Zhu	Radiomics	/	CTA	83	72	56.0 (51.0, 65.0)	65.0 (54.3, 73.0)	72	83
Zhu	Radiomics-morphological		CTA	83	72	56.0 (51.0, 65.0)	65.0 (54.3, 73.0)	72	83
Zhu	Clinical-radiomics-morphological		CTA	83	72	56.0 (51.0, 65.0)	65.0 (54.3, 73.0)	72	83
Zhu	Radiomics	/	CTA	43	32	57.9 (11.9)	67.5 (11.3)	34	41
Zhu	Radiomics-morphological		CTA	43	32	57.9 (11.9)	67.5 (11.3)	34	41
Zhu	Clinical-radiomics-morphological		CTA	43	32	57.9 (11.9)	67.5 (11.3)	34	41

### 3.2 Quality assessment of included studies

**Figure 2:**
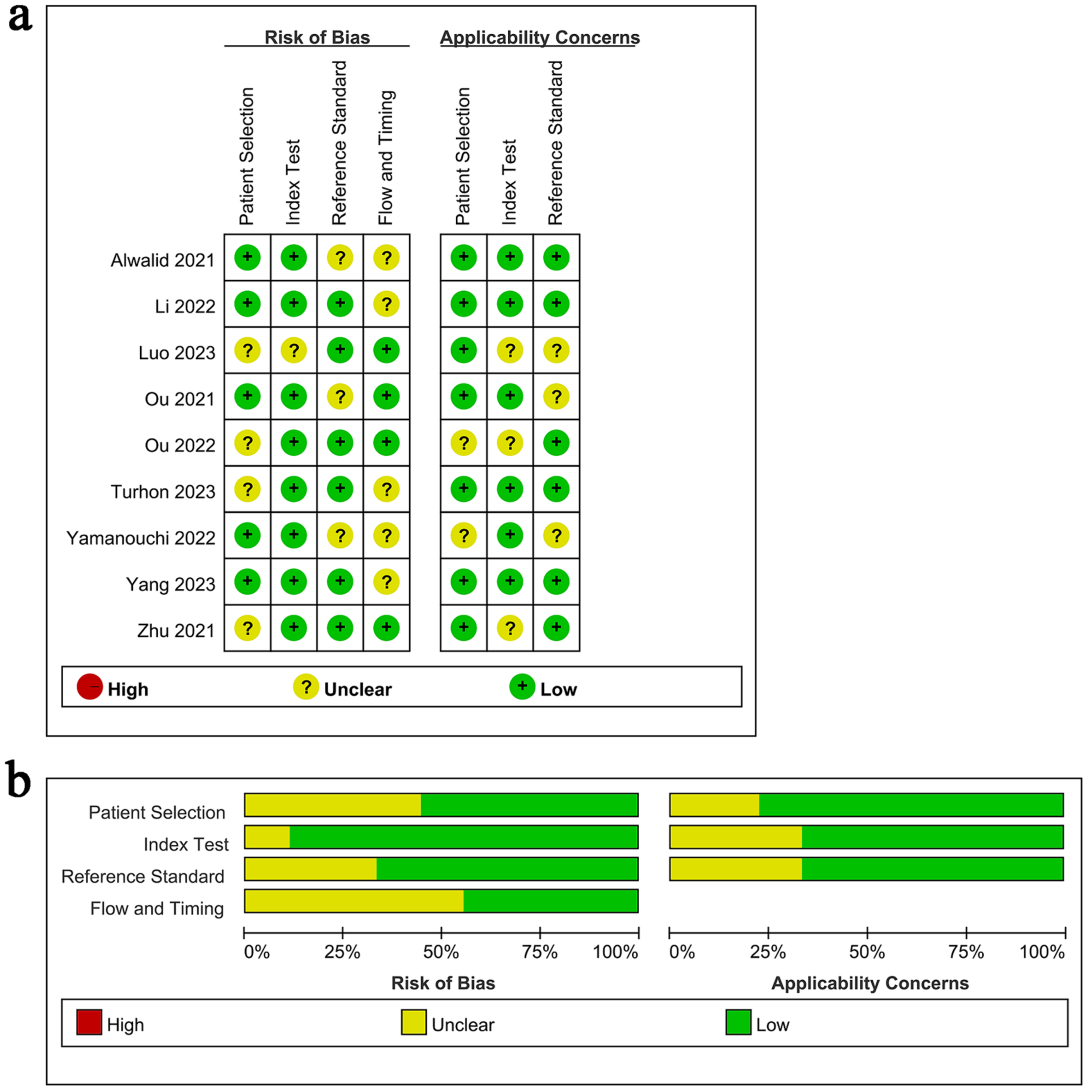
Risk of bias and applicability concerns summary.

The QUADAS-2 tool was used to assess the risk of bias and the RQS was used to assess the quality of each included study. Figure 2 presents the methodological quality evaluation results for the included studies. Overall, the included studies showed high quality, with most exhibiting low or unclear risk of bias. Detailed RQS of each study was presented in Supplementary Table S1.

### 3.3 Meta-analysis results

**Figure 3:**
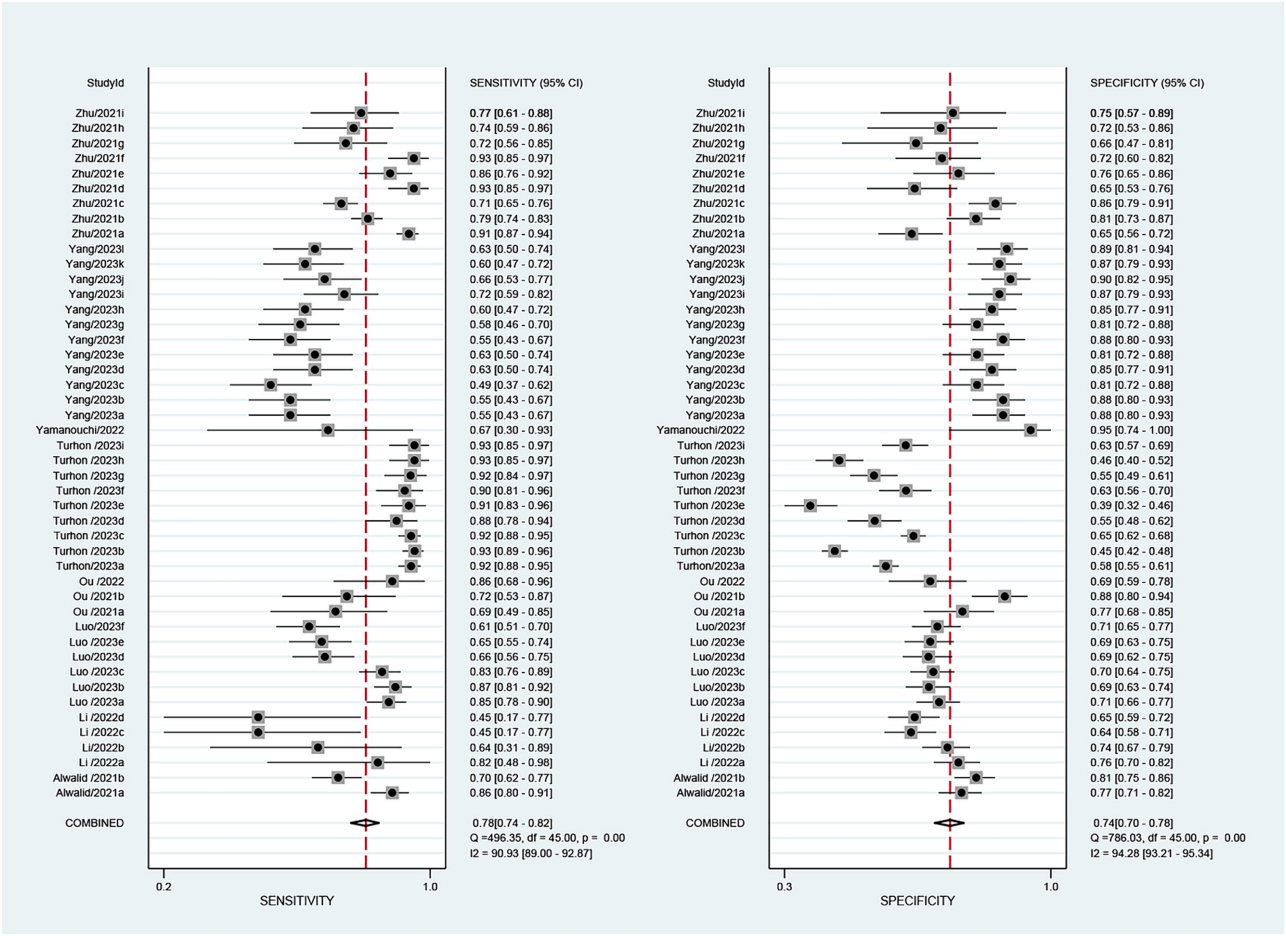
The forest map of the combined sensitivity, specificity of the predictive value of radiomics for intracranial aneurysm rupture.

**Figure 4:**
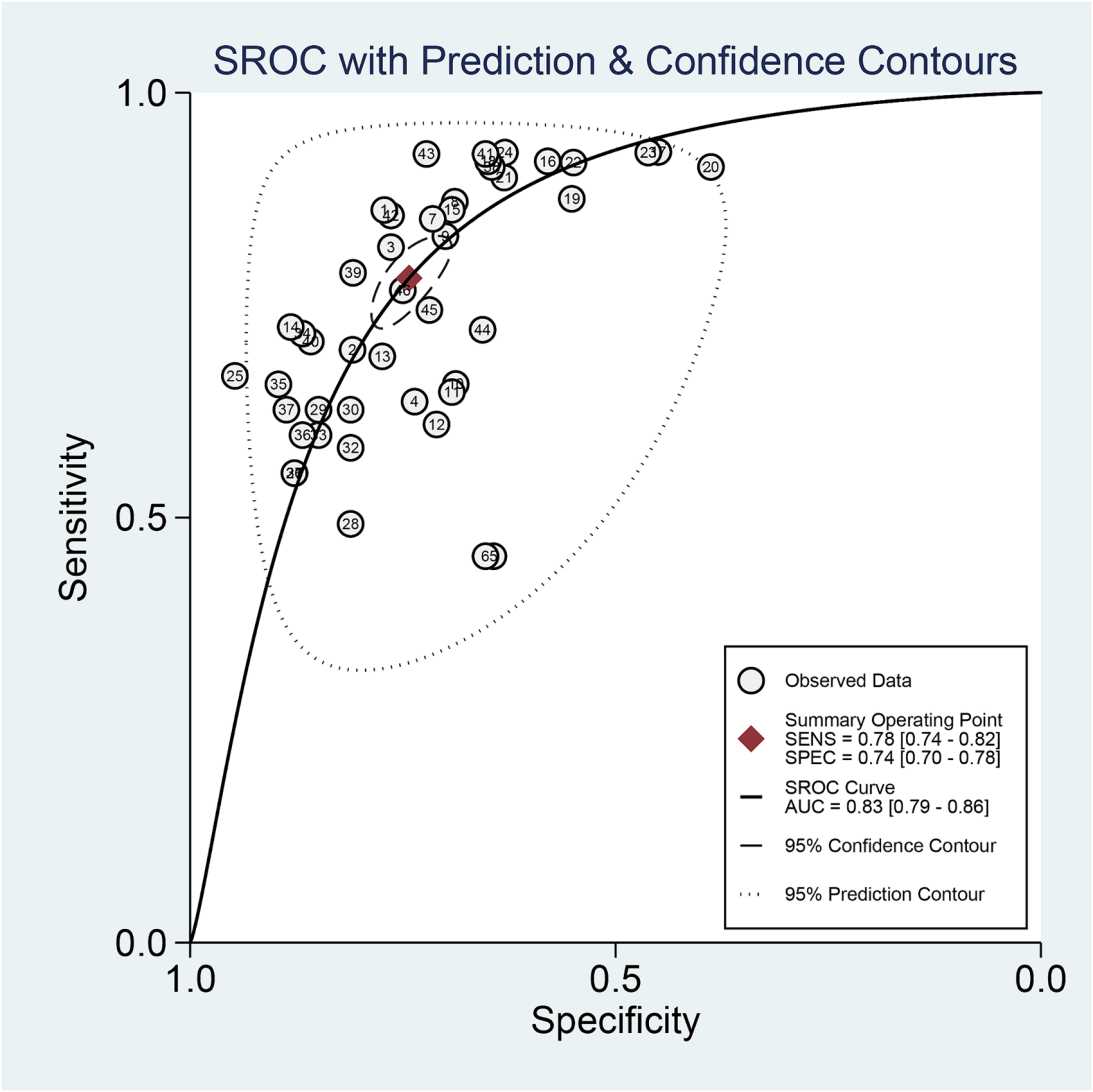
SROC curve of radiomics for predicting intracranial aneurysm rupture.

The 9 included studies reported 4,284 patients, with 1,411 patients having intracranial aneurysm rupture, resulting in a prevalence of 32.9%. The overall performance of radiomics for predicting intracranial aneurysm rupture showed a combined Sen of 0.78 (95% CI: 0.74–0.82), Spe of 0.74 (95% CI: 0.70–0.78), +LR of 3.0 (95% CI: 2.7–3.4), −LR of 0.29 (95% CI: 0.25–0.35), and DOR of 10 (95% CI: 9–12) (Figure 3). The overall forest plot revealed significant heterogeneity in both sensitivity and specificity, with *I*^2^ = 90.93% (95% CI: 89.00–92.87%) for Sen and *I*^2^ = 94.28% (95% CI: 93.21–95.34%) for Spe (Figure 3). Figure 4 presented the SROC with prediction and confidence contours, yielding an AUC value of 0.83 (95% CI: 0.79–0.86).

### 3.4 Publication bias

**Figure 5:**
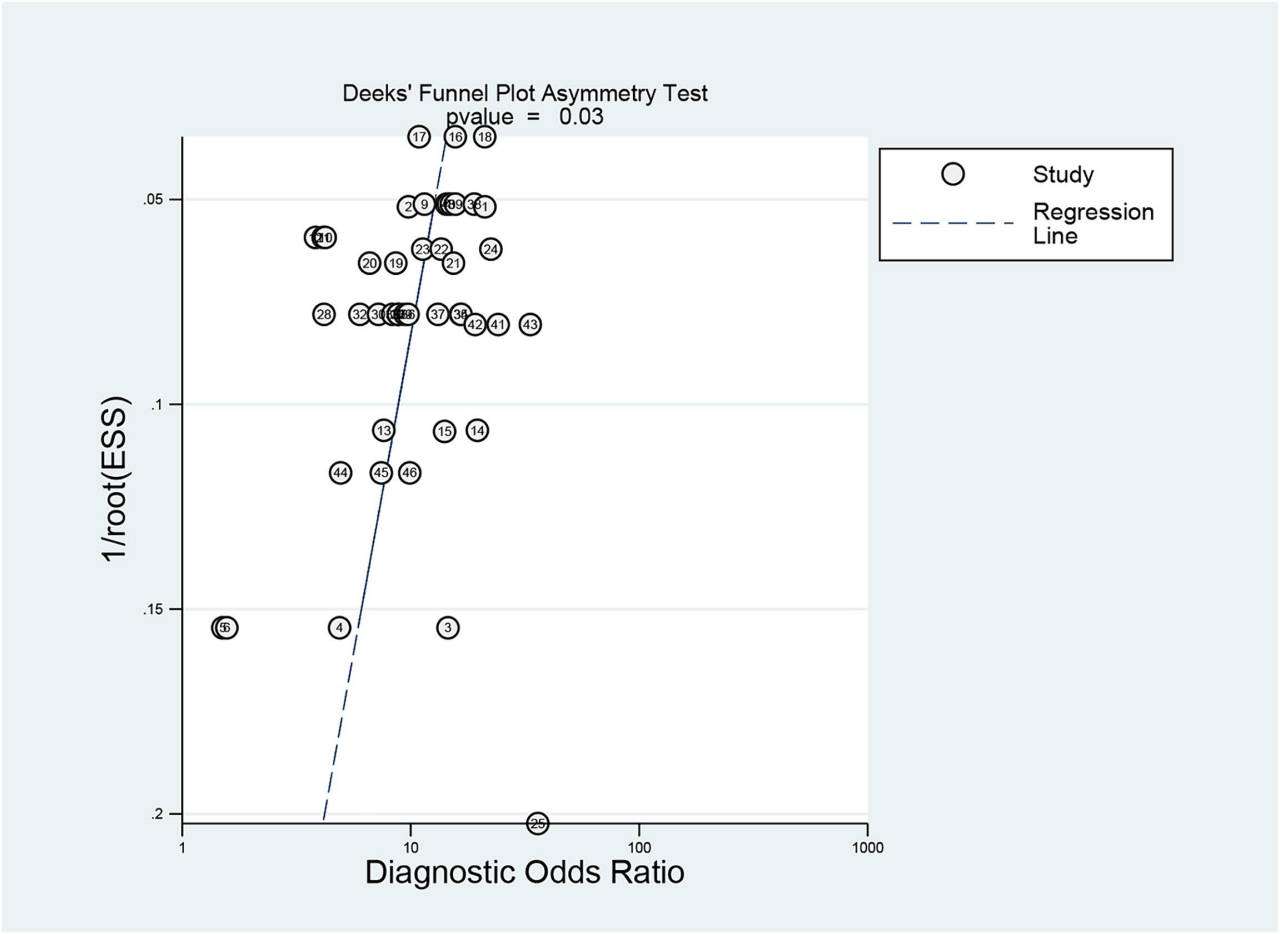
Detection of publication bias in predicting intracranial aneurysm rupture using radiomics.

**Figure 6:**
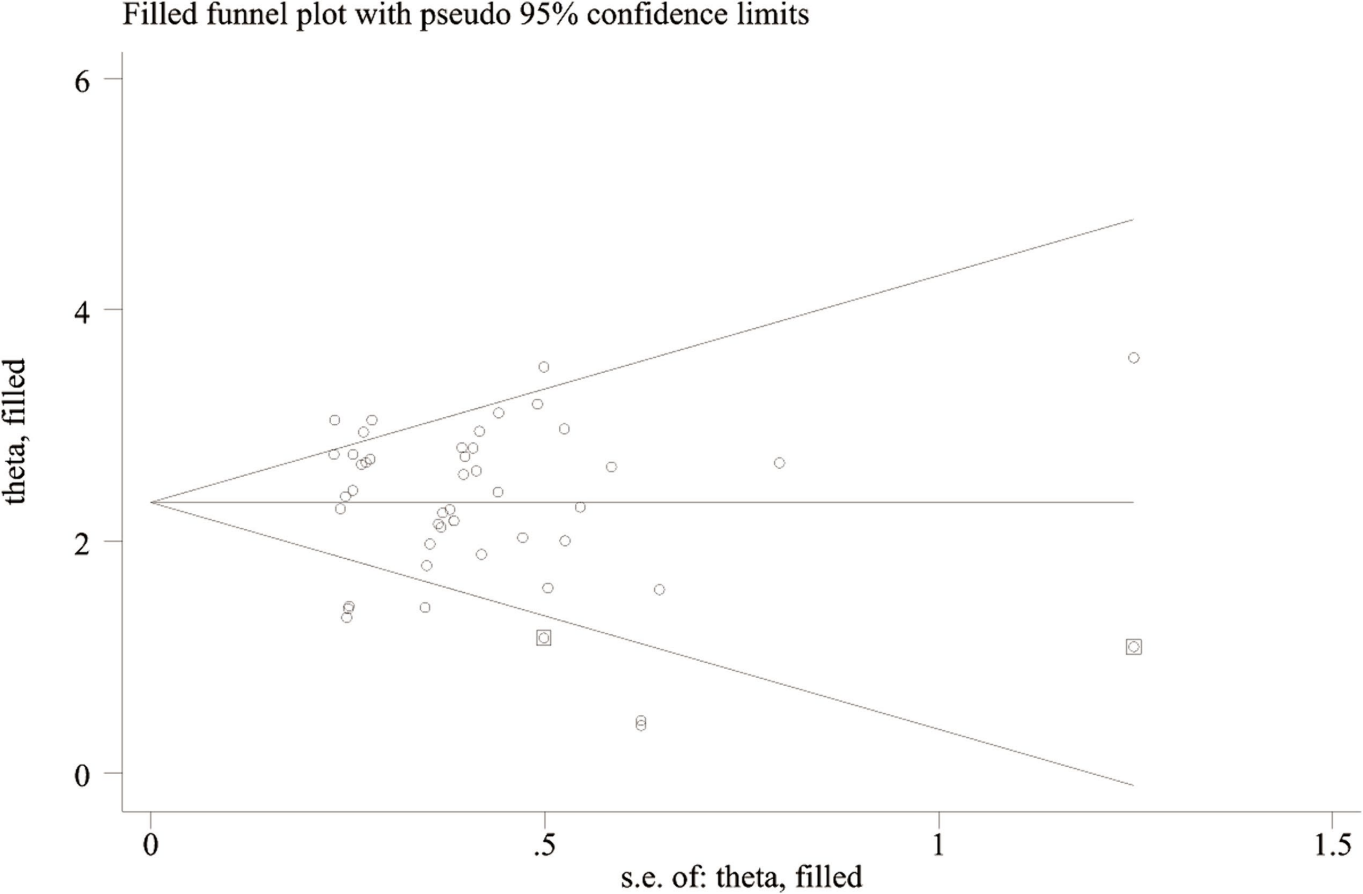
Funnel plot of “trim-and-fill method”.

Deeks’ test was employed to assess publication bias in the included studies, yielding a *p* value of 0.03. As the *p* value was less than 0.05, it suggested that this meta-analysis was affected by significant publication bias (Figure 5). After the number of missing published papers was filled by the “trim-and-fill method,” the results showed that the values of the corresponding variables did not change significantly before and after filling, suggesting that publication bias did not affect the overall effect size of the merger (Figure 6).

### 3.5 Post-test probability

**Figure 7:**
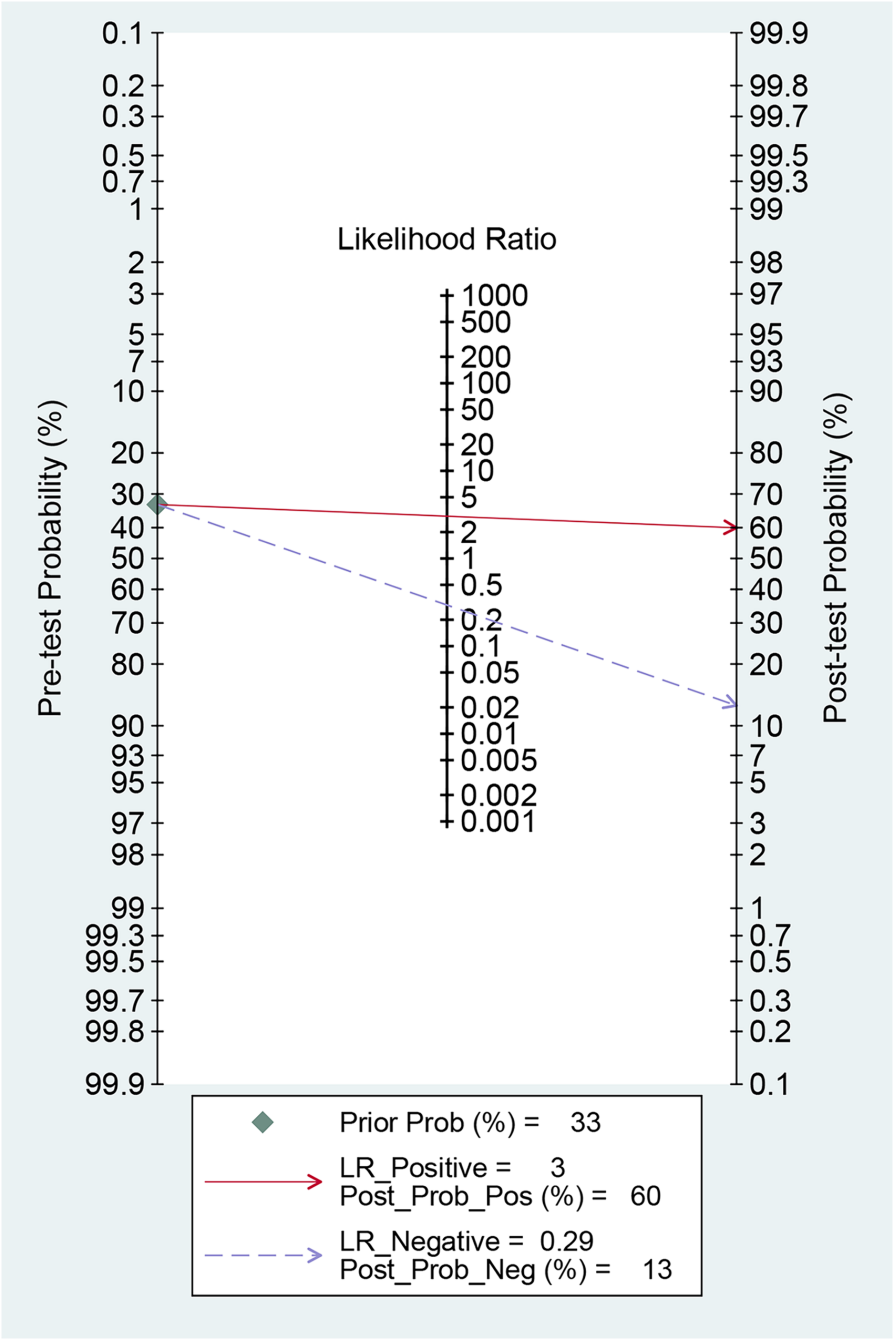
Fagan’s Nomogram for predicting intracranial aneurysm rupture using radiomics.

Before the application of radiomics, the pre-test probability of intracranial aneurysm rupture was 33%. The post-test probability of diagnosing ruptured intracranial aneurysms with positive radiomics results was 60%, while negative radiomics results yielded a post-test probability of 13%. When radiomics was positive, the prevalence of ruptured intracranial aneurysms was 60%, and when radiomics was negative, the prevalence was 13% (Figure 7).

## 4 Discussion

Radiomics and traditional morphological features of intracranial aneurysms are both derived from CTA images but differ in their biological significance and analysis methods. Radiomics features describe the shape and texture characteristics of intracranial aneurysms from a microscopic perspective (Collins et al., 2015; Xu et al., 2019), while traditional morphological features reflect the macroscopic observation. Radiomics features are three-dimensional, high-throughput biomarkers, but lack biological interpretability. Despite being based on two-dimensional image measurements and containing relatively little information, traditional morphological features remain an important tool for evaluating intracranial aneurysm stability in clinical practice due to their operability and intuitiveness. Numerous studies support the complementary role of combining radiomics and traditional morphological features.

This study combined existing clinical data on the use of radiomics to predict intracranial aneurysm rupture through a systematic review and meta-analysis, obtaining the combined diagnostic efficacy to comprehensively evaluate the predictive value of radiomics. The results suggest that radiomics can be used as an additional tool in assessing the rupture risk of intracranial aneurysms in clinical practice, with a combined Sen of 0.78, Spe of 0.74, +LR of 3.0, −LR of 0.29, and DOR of 10. However, considering the significant publication bias in the study conclusions, further research is needed to confirm the predictive value of radiomics for intracranial aneurysm rupture.

Machine learning, a subfield of artificial intelligence, can identify and process complex relationships between multiple variables in large data sets through computer algorithms, applying them to predict unknown data. Yang et al. (2023) demonstrated that 12 machine learning models established using radiomics features could predict the risk of intracranial aneurysm rupture in 576 cases (AUC: 0.713–0.889), with varying predictive performance among algorithms. The ensemble learning algorithm, particularly the adaptive enhancement model, exhibited the best predictive performance, with an AUC of 0.889 (95% CI: 0.842–0.936) in the training set. When the validation set underwent 3-fold cross-validation, repeated 5 times, the AUC of the adaptive enhancement model ranged from 0.842 to 0.918. Turhon et al. (2023) analyzed clinical data and DSA images of 1740 patients with intracranial aneurysms, confirming the importance of radiomics in assessing aneurysm rupture risk. The deep learning model, constructed by combining clinical, morphological, and imaging features, significantly outperformed the machine learning model (AUC: 0.878, 95% CI: 0.840–0.916) and the traditional Logistic regression model (AUC: 0.849, 95% CI: 0.808–0.890) in predicting aneurysm rupture (AUC: 0.929, 95% CI: 0.893–0.965; all *p* < 0.01). The deep learning model’s performance was also verified in internal and external data.

Ou et al. (2022) proposed a self-supervised and pre-trained deep learning model to predict the rupture risk of unruptured intracranial aneurysms in a follow-up of 2 years or more. The morphologically aware deep embedding method outperformed the deep learning model trained from scratch and the traditional morphological and imaging genomics models in predicting aneurysm rupture. The study also developed a computer-assisted risk assessment system based on the model. Preliminary tests by 5 neurosurgeons showed that the system could improve their ability to predict intracranial aneurysm rupture and assist in making clinical decisions based on case reasoning. This study overcame the influence of morphological changes before and after aneurysm rupture, enabling the training of deep neural networks with limited data, and has the potential to become an auxiliary tool for clinical intracranial aneurysm rupture risk assessment. Feng et al. (2023) first applied a three-dimensional convolutional neural network for automatic detection, segmentation, and morphological measurement of intracranial aneurysms on CTA. Morphological and radiomic features related to aneurysm rupture were input into machine learning models (support vector machines, random forests, and multilayer perceptrons) for training, resulting in a well-performing model on the training and test sets (AUC: 0.85–0.90). This method can automatically analyze CTA images of intracranial aneurysms and evaluate their rupture status within 1 min, improving clinical work efficiency. Radiomics demonstrates great application potential in assessing intracranial aneurysm stability, and combining machine learning, particularly deep learning, with radiomics is expected to enhance the ability to predict intracranial aneurysm stability.

This study has several limitations. First, most of the included studies are retrospective, which may have potential uncontrollable bias risks. Second, the majority of studies are from Asia, and further research is needed to confirm whether the predictive value of radiomics for intracranial aneurysm rupture can be extended to other regions or countries. Additionally, the numerous and scattered types of radiomics models involved in this study contribute to the significant overall heterogeneity. However, due to data limitations, subgroup analysis based on the model to explore potential sources of heterogeneity could not be performed. The lack of biological interpretability of radiomics is also one of the limitations of this paper, and in order to address the challenge of biological interpretability, future research can focus on combining radiomics with genomic or molecular data to improve the interpretability and clinical relevance of models. Lastly, some studies included in this meta-analysis contained images from external validation populations, obtained by different scanning parameters and machines, which may affect the results and heterogeneity of model validation. In the future, more research is needed to explore the radiomics of different types of aneurysms and to develop models that combine clinical data with radiomics while testing these models in real-world clinical scenarios.

## 5 Conclusion

Although the results of this study suggest that radiomics can be used as an auxiliary tool to improve the diagnostic efficacy of ruptured intracranial aneurysms to some extent, the quality of the evidence is limited by significant heterogeneity, potential publication bias, small sample size, and retrospective study design. In the future, more large-scale, prospective, multicenter clinical studies are needed to further evaluate the value of radiomics in predicting intracranial aneurysm rupture.

## Data Availability

The original contributions presented in the study are included in the article/Supplementary material, further inquiries can be directed to the corresponding authors.
