# Supplementary material for: Predictive value of radiomics for intracranial aneurysm rupture: a systematic review and meta-analysis
Source: Front Neurosci. 2024 Oct 9;18:1474780. doi: 10.3389/fnins.2024.1474780 (PMC11496283; doi:10.3389/fnins.2024.1474780)
Supplement: Supplementary file 1 [file Table_1.DOCX]

| Supplementary Table S1. The Radiomics Quality Score (RQS) for each included study. | | | | | | | | | | | | | | | | | |
| --- | --- | --- | --- | --- | --- | --- | --- | --- | --- | --- | --- | --- | --- | --- | --- | --- | --- |
| Study | Image protocol | Multiple segmentations | Phantom study | Multiple timepoints | Feature radiomics | Non-radiomics | Biological correlates | Cut-off | Discrimination/resampling | Calibration/resampling | Prospective | Validation | Gold standard | Clinical utility | Cost | Open science | Total |
| Alwalid 2021 | 1 | 1 | 0 | 0 | 3 | 0 | 0 | 0 | 2 | 0 | 0 | 3 | 0 | 2 | 0 | 0 | 12 |
| Li2022 | 2 | 1 | 0 | 1 | 3 | 1 | 1 | 0 | 2 | 0 | 0 | 5 | 0 | 2 | 0 | 0 | 18 |
| Luo 2023 | 0 | 0 | 0 | 0 | 3 | 0 | 0 | 0 | 2 | 0 | 0 | 5 | 0 | 2 | 0 | 0 | 12 |
| Qu 2021 | 0 | 1 | 0 | 0 | 3 | 1 | 0 | 1 | 2 | 0 | 0 | 4 | 0 | 2 | 0 | 0 | 16 |
| Qu 2022 | 0 | 0 | 0 | 0 | 3 | 1 | 0 | 0 | 2 | 0 | 0 | 4 | 0 | 2 | 0 | 0 | 12 |
| Turhon 2023 | 1 | 1 | 0 | 1 | 3 | 1 | 1 | 1 | 2 | 2 | 0 | 2 | 0 | 2 | 0 | 0 | 17 |
| Yamanouchi 2022 | 1 | 1 | 0 | 1 | 3 | 1 | 0 | 1 | 2 | 2 | 0 | 2 | 0 | 2 | 0 | 0 | 16 |
| Yang 2023 | 1 | 0 | 0 | 0 | 3 | 1 | 0 | 0 | 2 | 2 | 0 | 0 | 0 | 2 | 0 | 0 | 11 |
| Zhu 2021 | 1 | 1 | 0 | 0 | 3 | 1 | 0 | 1 | 2 | 2 | 0 | 2 | 0 | 2 | 0 | 0 | 15 |
